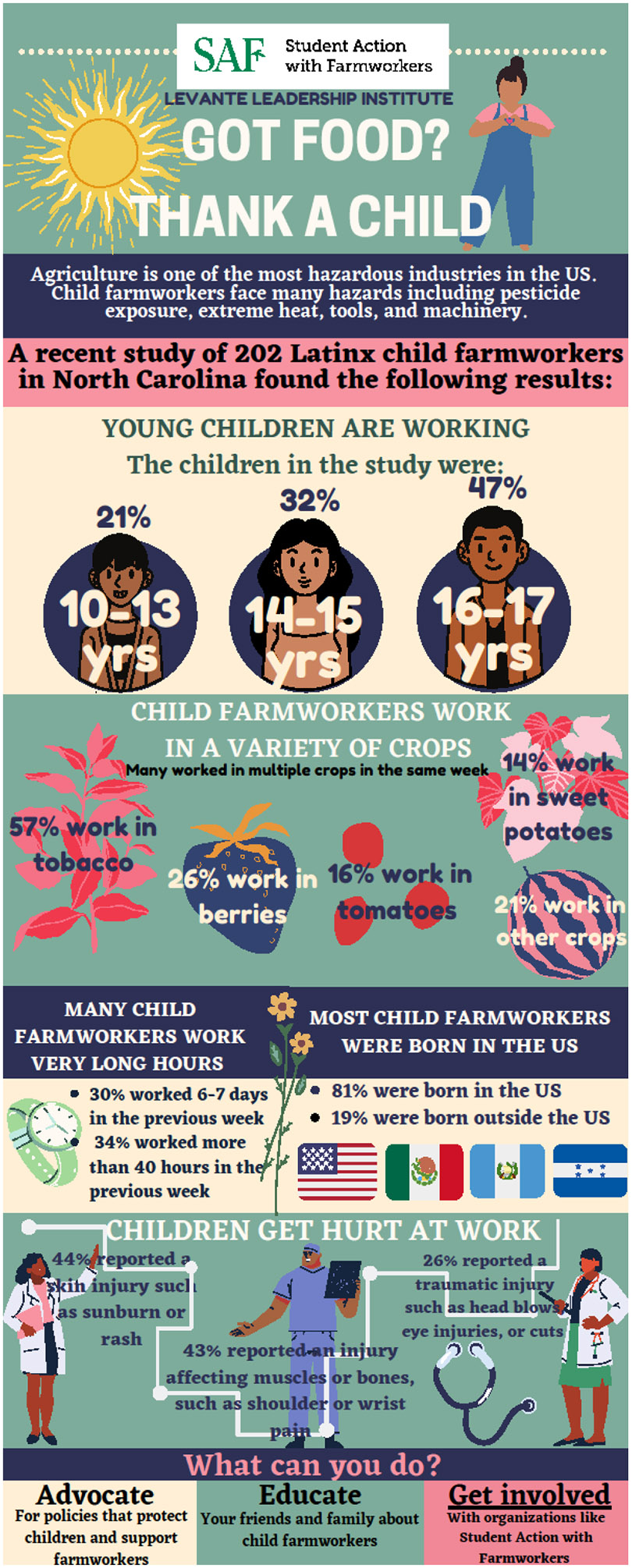# Disseminating Research Results to Raise Awareness About Child Farmworker Health Through Youth-Driven Participatory Infographics

**DOI:** 10.35844/001c.159444

**Published:** 2026-06-08

**Authors:** Taylor J. Arnold, Maria Juarez, Ramon Zepeda, Vanessa Gonzalez, Elber Lopez Paz, Fidel Jaramillo-Plata, Sylvia Zapata, Sara A. Quandt, Thomas A. Arcury, Stephanie S. Daniel

**Affiliations:** 1 Department of Family & Community Medicine, Wake Forest University School of Medicine; 2 Student Action with Farmworkers; 3 Virginia Interfaith Center for Public Policy; 4 Sampson Community College; 5 Department of Epidemiology and Prevention, Wake Forest University School of Medicine

**Keywords:** Community-Based Participatory Research, Youth Participatory Research, Child Farmworkers, Health Equity, Participatory Dissemination

## Abstract

Despite increased emphasis on disseminating research results beyond academic outlets, effective dissemination to non-academic audiences remains a challenge. We describe a collaborative, youth-driven participatory approach to developing infographics for sharing research results from a study on the health and safety of hired Latine child farmworkers in North Carolina. Academic investigators partnered with rural youth from farmworker families participating in a youth leadership program. We detail the step-by-step infographic development and dissemination process and reflect on lessons we learned throughout the partnership. This approach demonstrates the potential for adapting youth-driven infographic development processes to diverse research and practice settings, ultimately increasing the utility and uptake of research findings in community settings.

Despite substantial investments of time, money, and resources in health equity research, gaps remain in understanding best practices for disseminating research results effectively outside of traditional academic outlets (Long et al., 2017). In recent years, both researchers and funding agencies have placed more emphasis on returning results to research participants and communities (Cunningham-Erves et al., 2021; Korfmacher & Brody, 2023; Thompson et al., 2017). New developments in translational and dissemination science show promise in developing priorities and best practices for disseminating research (Gustavson et al., 2025; Mayo-Gamble et al., 2022). Yet, researchers often continue to struggle with effectively communicating their results to lay audiences (Long et al., 2017). Such communication requires managing the time and logistical barriers of dissemination beyond traditional academic publishing (Long et al., 2019), incorporating community members into the process (Chen et al., 2010) and dealing with the lack of academic incentive structures that reward these efforts (Gustavson et al., 2025).

One practical way to ensure that health research results are disseminated appropriately is to involve community members and stakeholders throughout the entire research process, from conceptualization through dissemination (Wallerstein & Duran, 2010). There are many established approaches for conducting community-engaged research (CEnR), including Community Based Participatory Research (CBPR), Participatory Action Research (PAR), and Youth Participatory Action Research (YPAR) (Haapanen & Christens, 2021). While these approaches differ in orientation and strategy, they share the common goal of ensuring equitable partnerships with communities and an ethical imperative of ensuring that research results are shared with communities in ways that can benefit the community (Arcury & Quandt, 2020; Israel et al., 1998).

Youth-partnered research approaches offer many opportunities for enhancing research but also present unique challenges. Youth often face greater barriers to participation related to power differentials when working alongside adult researchers (Cammarota & Fine, 2010; Mackey et al., 2021; Ozer, 2016). Authentic community-based research with young people requires significant time and may be constrained due to limited resources and time for both researchers and youth (Kim, 2016). Additionally, sustaining youth-academic partnerships can be difficult, due to the cyclical nature of youth moving in and out of youth-oriented programming during life transitions (Ozer et al., 2013; Yoder et al., 2025). Despite the inherent challenges in conducting youth-partnered research, effective partnerships can bring many benefits for researchers, youth, and communities. Researchers benefit from the insight and expertise that youth bring when their input is valued, increasing the feasibility, effectiveness, and ecological validity of the work (Hawke et al., 2018). Youth benefit from partnering with researchers by building research, leadership, and advocacy skills, while networking with professionals and gaining experience in collaborative teams (Johnson Gray & Ren, 2024; Smith et al., 2024). Further, YPAR in particular can be viewed as a pedagogical approach to empowerment, where youth draw from their own experiences to develop strategies to challenge, resist, and bring awareness to unjust systems affecting youth (Anderson et al., 2024; Cammarota & Fine, 2010; Ozer et al., 2020; Teixeira et al., 2021). Finally, the *process* of participating in collaborative research during a critical developmental window of opportunity can provide meaningful ways for youth to contribute to their communities (Ballard & Syme, 2016).

Some resources are available to guide researchers in dissemination strategies outside of traditional academic outlets (CARE: (Community Alliance for Research and Engagement, n.d.); VICTR: (Vanderbilt Institute for Clinical and Translational Research, n.d.)). Strategies include drafting lay summaries, press releases, policy briefs, blog posts, infographics, and visual/video abstracts, and participating in non-academic gatherings (Ross-Hellauer et al., 2020). Infographics are visual representations of information or data that can simplify complex topics in a brief and comprehensible way (McCrorie et al., 2016). Infographics are well-suited for dissemination on social media platforms, thus increasing the visibility of research to academic and non-academic audiences alike (Huang et al., 2018; Jeyaraman et al., 2025; Kunze et al., 2021). Infographics are particularly useful for CBPR with youth because of the limited text, graphical depictions of findings, digital format, and visual appeal, which enhance their value to community-based and advocacy organizations (Nelson Ferguson et al., 2025; Quandt et al., 2023).

## Objectives

The objectives of this manuscript are: (1) to describe our collaborative, youth-driven process for developing infographics to disseminate research study findings to communities; and (2) to reflect on the lessons we learned through the process with attention to adapting our approach to a broad range of research and practice settings.

## Study and Partnership Overview

### Hired Child Farmworker Study

Agriculture is one of the most hazardous industries, and workers of all ages experience high rates of injury, illness, and fatality compared to other industries (Mulhollem, 2021). Despite this fact, many in the general public are unaware that children younger than 12 years old are legally permitted to work in US agriculture due to legislation dating back to the late 1930s (Wage and Hour Division, U.S. Department of Labor, 2016; Wiggins, 2020). The Hired Child Farmworker Study (2016-2022) used a CBPR approach and a multi-method design to investigate the health and safety of hired Latine child farmworkers in North Carolina (NC) (Arcury, Arnold, Sandberg, et al., 2019). The study design included a formative qualitative component with 30 Latine child farmworkers, followed by a three-year quantitative component with 202 child farmworkers (62.4% boys and 37.6% girls) ages 10-17 at baseline, with repeated survey, environmental exposure, and clinical measures. Findings documented that children working in agriculture experienced a poor work safety environment (Arcury, Arnold, Mora, et al., 2019; Arcury et al., 2020; Quandt et al., 2019) coupled with high rates of occupational injury (Arcury, Arnold, Quandt, et al., 2019), environmental exposures (Arcury et al., 2021; Arnold et al., 2020, 2024) and compromised educational experiences (Quandt et al., 2021). Our CBPR model for the study incorporated community participation in four domains: (1) consultation — delineating what should be done; (2) strategic planning — deciding how it should be done; (3) implementation — completing the actual tasks; and (4) dissemination — sharing what was accomplished with the community and policy makers (Arcury & Quandt, 2014; Quandt et al., 2001).

### Youth Co-Investigators

One critical component of the study’s CBPR design was partnering with youth who had farm work experience. To do so, we collaborated with a non-profit organization, Student Action with Farmworkers (SAF), with whom the core investigators had partnered for other farmworker research for over 20 years (Arcury et al., 2013; Arcury, Jensen, et al., 2017; Liebman et al., 2013; Quandt et al., 2013). One of *SAF’s* programs is the *Levante* Leadership Institute (hereafter: *Levante* program), which is an academic year program that annually involves 10-15 rural youth from farmworker families in eastern NC. The *Levante* program equips high school students from farmworker families with resources on college preparation, life skills, cultural arts, and advocacy. The partnership between the research team and the *Levante* program involved multiple cohorts of youth serving as study co-investigators (2 each year) and a youth advisory board (remainder of students participating in the *Levante* program). Youth co-investigators were selected by the *Levante* Youth Director and academic Project Manager after initial conversations about the roles with the entire group to gauge interest and commitment. Youth co-investigators took a leadership role by meeting more frequently with the research team to plan activities and agendas for quarterly youth advisory board meetings, troubleshooting emergent research needs, participating in manuscript development, and attending and presenting at conferences. To acknowledge their leadership roles and time investments, the 2 youth co-investigators each received a $500 stipend per semester. The partnership was mutually beneficial to the academic investigators (e.g., grounding the research questions, study materials, and approach in the lived experience of youth from farmworker families) and *Levante* program youth (e.g., building research, advocacy, leadership, and professional skills). We have described the overall structure of the partnership and roles of the youth advisors in detail elsewhere (Arnold et al., 2019). The present manuscript was developed with former and current staff and youth from the *Levante* program and academic investigators from the Hired Child Farmworker Study.

## Youth-Driven Collaboration Process and Infographic Development Steps

From 2019-2021, through a series of iterative workshops (approximately 1-2 hour sessions during the *Levante* program’s monthly meetings), the study team completed hands-on activities to brainstorm potential dissemination strategies. Our general and open-ended goal, as we described to the youth, was to “share some results of the study in a creative way.” The study team planned and co-led activities with the 2 youth co-investigators. During sessions, we completed adapted versions of free listing and pile sorting activities (Frasso et al., 2018; Weller & Romney, 1988) in which students were asked to list ideas to share the study findings and to rank order by sorting them into piles. These hands-on activities facilitated the discussion of different ways to share information including documentaries, skits/performances, songs, and infographics. The group achieved consensus that infographics would be the most effective way to disseminate research findings within the constraints of youth leadership program’s programmatic activities (i.e., the research collaboration was a limited portion of their overall activities).

### Step 1: Envisioning the Audience and Purpose

**Figure 1.**
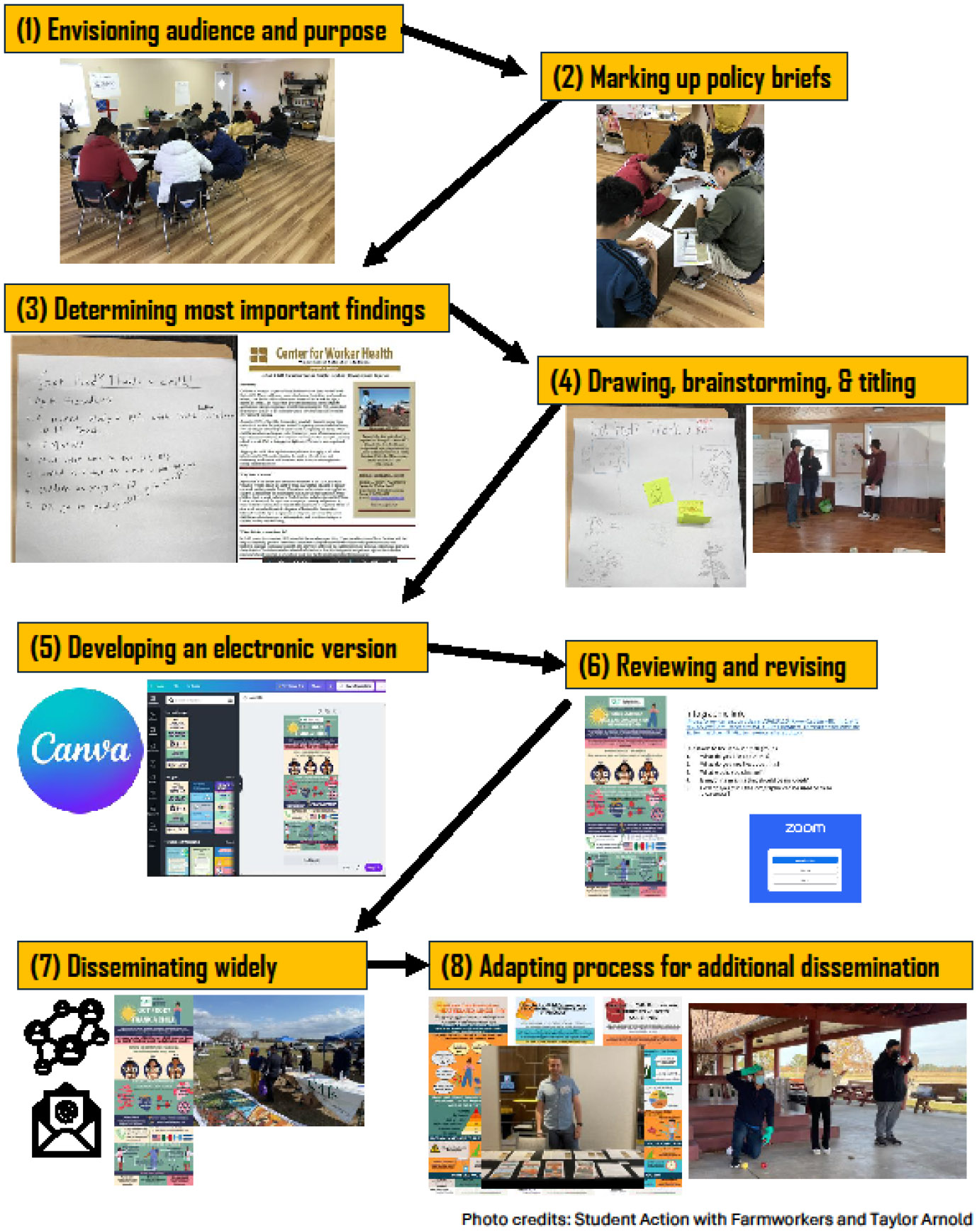
Youth-Driven Infographic Development Steps

Upon deciding to collectively work on an infographic as the primary dissemination method, we worked together to define the audience and purpose of the infographic (Figure 1). During one of the early workshops, we completed activities exploring how research can be used for advocacy and connected this to the goal of improving the health of child farmworkers. The *Levante* youth envisioned sharing the infographic in public places such as community stores or schools, as well as in digital formats online. They shared their perception that the general public, including many of their peers, were unaware that children worked in agriculture, that it was dangerous, or that it was legal. Therefore, they decided that the general purpose of the infographic was to “raise awareness” and educate the community about child farmworkers with the goal of increasing action and advocacy around this issue.

### Step 2: Reviewing and Marking up Policy Briefs from Published Study Manuscripts

The next step was to decide on the content and focus area of the infographic. As part of our study team’s CBPR approach and longstanding partnerships, we had an established practice of developing “policy briefs” from manuscripts, in order to ensure that research findings were accessible to community organizations and policy makers (Arcury, Wiggins, et al., 2017). Following a standardized template, the policy briefs are 2-page (front and back) summaries of peer-reviewed, published manuscripts that specify what the researchers did, what they found, why it is important, and recommendations. They include brief paragraph text, bulleted text, and graphics such as bar graphs or pie charts. After discussing with *Levante* co-investigators, we decided that policy briefs would provide a good medium for deciding the content for infographics, rather than asking *Levante* program students to review entire scientific manuscripts (though full versions were also made available to the group). We chose 2 policy briefs including one that covered baseline descriptive information about the sample of child farmworkers, and one that covered occupational injury characteristics. During workshop sessions, *Levante* program youth split into smaller teams. We asked them to mark up the policy briefs to highlight things they found interesting, important, or surprising for the intended audience and purpose.

### Step 3: Determining the Most Important Findings to Include for the Audience

Using marked up policy briefs, we collectively narrowed the results to the key findings that would make an impactful infographic. The *Levante* program youth shared what they had marked up on the policy briefs and why they felt the findings should be included on the infographic. At the end of this discussion, we had a list of findings deemed important to raise awareness about child farmworkers. We then completed a ranking activity to narrow down the list to 5 key findings in addition to some of the context for the study. The 5 key findings related to the age ranges of study participants, types of crops they worked in, work schedules, nationalities, and reported injuries.

### Step 4: Titling, Brainstorming, and Drawing Ways to Depict the Findings

Once we decided on key findings, we focused on brainstorming ways to depict the findings and on titling the infographic. In small teams, *Levante* program youth were tasked with developing the infographic on large flip-chart paper and then presenting their ideas to the group. They also pitched ideas for how to name the infographic itself. There were differing opinions on the title, which we used as an opportunity for healthy debate, voting, and consensus building. Eventually, the group landed on “Got food? Thank a child!” The name is a creative adaptation of one of SAF’s campaigns “Got food? Thank a farmworker.”

### Step 5: Developing an Electronic Version of the Infographic with a Summer Intern

After finalizing the infographic content and title, we hosted a summer intern from one of SAF’s programs for college students (The Into the Fields Program). Due to the COVID-19 pandemic in the summer of 2020, we had to pivot to a remote internship where the intern worked to develop policy briefs and the *Levante* program youth infographic. The study team and intern met virtually with *Levante* co-investigators, who shared pictures of their drawings and brainstorming sessions, and the overall purpose of the infographic. The intern drafted the first version of the infographic using Canva ©, an online graphic design software (https://www.canva.com). The intern and study team members met virtually multiple times throughout the process with *Levante* co-investigators in order to share ideas and make revisions based on the youth co-investigators’ feedback. The *Levante* co-investigators provided substantive direction and vision for the first complete draft, including the order and depiction of findings, selection and style of graphics, and the bright color scheme.

### Step 6: Reviewing and Revising the Infographic

With a complete draft available, the study team held an online meeting with *Levante* program youth (many who had been involved in the development from the beginning). During the session, we discussed the process of developing the infographic and shared an electronic version. In breakout rooms, students were asked to consider the following prompts: “What do you like? What do you not like? What would you change? What is missing? How can this be used to raise awareness?” As a group, we came back together to discuss the feedback and make edits. One notable change we made based on this feedback was a call to action, which the youth felt was important to meet the infographic’s original goal of “raising awareness.” At the bottom of the infographic, we added a section titled “what can you do?” that included three simple and attainable actions: 1) Advocate for policies that protect children and support farmworkers, 2) Educate your friends and family about child farmworkers, and 3) Get involved with organizations like SAF. Once we achieved consensus on the final version of the infographic, a certified translator translated it into Spanish, and we asked specific *Levante* program youth students and another certified translator to review and provide feedback on the translation.

### Step 7: Disseminating the Infographic Widely

We shared electronic and physical copies of the infographic (Figure 2) in multiple digital and in-person venues. Due to the 2021 release date and COVID-19 restrictions for public gatherings, we initially shared the infographic in a digital format. Outlets included broad local coalitions such as the Farmworker Advocacy Network (FAN), made up of organizations serving farmworkers in health, advocacy, legal, education, and religious domains. Many of these organizations shared the infographic through their own newsletters and broad networks. We also shared the infographic with the Child Labor Coalition (CLC) ( https://stopchildlabor.org/), which has an extensive domestic and international reach. As a demonstration of the reach and impact of the infographic, in contrast to more traditional publication in academic journals, we were invited to write a guest blog post featuring the infographic and discussing the findings for an NC education policy advocacy organization that was picked up by additional news outlets.

As COVID-19 safety protocols became normalized for gatherings in later 2021-2022, we distributed physical copies of the infographic in community spaces such as farmworker service organizations, by tabling at farmworker festivals and service provider meetings, and to organizations requesting copies. *Levante* program youth distributed infographics at drive-through food distribution events and health fairs for farmworker families experiencing lost income due to the pandemic. Through these events, they made new connections within the farmworker community while sharing research results, but also invited eligible farmworker youth who were attending the events to join the *Levante* program.

### Step 8: Using the Process Designed by the Youth to Develop Additional Infographics and Dissemination Strategies

The success and uptake of the initial infographic developed by *Levante* program youth led us to create additional infographics following a similar process covering specific research findings from other manuscripts and policy briefs the study team had published since the development of the first infographic. With the help of another summer intern, and a process and overall format and style in place, we developed 3 additional infographics focused on educational experiences of child farmworkers, musculoskeletal injuries, and heat-related illness (see supplemental material). *Levante* program youth provided feedback and direction through online and in-person workshops. We have described the process of creating additional infographics in detail in a different manuscript (Quandt et al., 2023).

The infographics also spurred additional creative dissemination strategies by the *Levante* program youth, as they used them as the basis for in-person popular theater workshops (Boal & McBride, 1993; Freire, 1973), a dynamic approach that SAF uses for outreach with farmworkers. The youth developed skits and popular theater activities to portray what they learned about issues affecting children working in agriculture such as heat stress and other injuries. They also used what they learned through the infographics to rewrite the lyrics of popular and culturally relevant songs to include messages to raise awareness about heat stress and then performed these for community audiences.

## Reflections and Lessons Learned

We learned several lessons throughout the process of developing participatory youth-driven infographics. Identifying these lessons may help make this process transferable or adapted to other community-based research contexts (with youth or adults).

### Maintaining Flexibility

Community-partnered research requires flexibility (Arcury & Quandt, 2014; Israel et al., 1998; Quandt et al., 2001). For youth-partnered research in particular, researchers must take a reflective look at their own priorities and demands versus the priorities and demands of youth. While academics are operating within their own institutional and personal contexts with time constraints, youth have their own pressures and demands on their schedules (Malorni et al., 2022). Often, due to school, extracurricular activities, and/or jobs, the availability of youth to participate in research is limited. Therefore, it is important for researchers to make themselves available and plan activities outside of “business hours,” including weekends. To increase attendance and reduce the time burden, it is useful to add research study activities to existing meetings, rather than planning additional meetings. In our study, many of our workshops to develop and refine the infographics with youth were in the evenings or during one of their *Levante* program retreats on weekends. Where feasible, investigators or supervisors should set early expectations for their staff to plan for and honor a flexible work schedule to account for night and weekend youth-partnered research (e.g., take off weekdays if working on weekends).

Flexibility is also required to prepare for and respond to the potential flux of both youth and community-based organization partner staff. Youth rotate in and out of programs due to their own schedules and commitments or aging out (e.g., graduating). Staff at community-based organization research partners may also rotate in and out as they pursue education or other opportunities. Therefore, researchers must be flexible in understanding that this is often the nature of the work. It requires constant assessment and re-assessment to ensure that new youth participants or staff leaders understand the history of the project and partnership and remain oriented to the shared collective goal. Yet, simultaneously, it is important to avoid alienating incumbent youth by too much repetition of the same information. Developing and utilizing a brief resource to orient new youth and staff to the project outside of full meetings can be helpful. While it can be difficult to maintain momentum and relationships amid personnel changes, remaining flexible and celebrating the accomplishments of youth and staff partners transitioning to achieve other goals outside of the research partnership is part of the process.

### Resources and Time

Youth-partnered researchers should allocate adequate resources and time for completing the community-partnered study activities. Anticipating these issues early can improve the outcomes of the work. To use our study (and other similar grant-driven timelines) as an example, the final year is often focused on dissemination. However, if possible and scientifically-sound, investigators should make effort to publish results earlier as the data become available, as it becomes exponentially more difficult after budgeted effort on a project ends. Part of the success of our infographics was that we published qualitative and baseline survey results, and developed the associated policy briefs, in year 3 of the 5-year study (which also received an extension to a sixth year). This allowed ample time to work with youth at a reasonable pace to develop the infographics during their already scheduled programming (meetings and retreats), while also covering other ongoing project needs (Arnold et al., 2019).

It is important to compensate youth for their time to acknowledge and diminish inherent power-differentials between academic investigators and youth partners (Jacquez et al., 2013). However, compensation for youth can create additional institutional hurdles and dilemmas (Kornbluh et al., 2025). Nevertheless, paying youth for their time is critical not only because it incentivizes participation, but, more importantly, it reflects a clear stance that their time and expertise are valued by the research team. Further, it may be necessary to allocate money for in-person travel to youth-partnered workshops. For our study, the research team is located around 2.5 hours driving distance from the rural location where *Levante* program meetings were held. However, in our experience, showing up to events (and staying beyond the specified workshop period) was generative in building and maintaining rapport with youth researchers. Linking back to the flexibility requirement, it is possible to adapt to online modalities (as we all were forced to do during the COVID-19 pandemic); however, it requires creativity to develop rapport and engaging activities. While virtual meeting formats are widely available and normalized in the years following the initial waves of the pandemic, hybrid meeting schedules with at least some in-person meetings are important for rapport and relationship building, engagement with the project, and for soliciting a greater breadth and depth of feedback in group settings.

### Strategies for Engagement and Shared Decision-Making

Developing strategies for engagement and shared decision-making are critical to ensure the project moves forward while ensuring everyone’s perspective is valued (Cammarota & Fine, 2010; Mackey et al., 2021; Malorni et al., 2022; Ozer, 2016). Offering multiple roles and modes of participation allows youth to self-select into activities that they find exciting. Having a smaller team of youth (in our case, 2 youth co-investigators) involved in planning the workshop agendas helps generate new ideas, filter out activities that may not work, and increase the chance of smooth functioning on the day of the workshop, especially when you are creating new activities that have not been tested before. Having youth lead as many of the activities as possible facilitates greater engagement and sets a different tone of openness with the broader youth group. In the latter phases of our infographic development, youth who were not “officially” paid co-investigators, but were just interested and invested in the process, continued having a more hands-on role in driving the design of the infographic outside of full workshop sessions. The role of having a summer intern from SAF take the lead on developing the infographic using online design software (https://www.canva.com) helped to ensure completion. Asking for feedback from the *Levante* co-investigators and members early and often on versions of the draft, specific graphics to use, and how to display the findings offered multiple modes of participation through verbal discussions and/or written feedback.

One approach that is particularly valuable and provides opportunities for many roles and shared decision-making is to ensure that workshops are centered around hands-on activities that incorporate movement, interaction, fun, and competition where possible. This is a practice within our own team that is youth-driven and aligned with one of our initial meetings with the group in which they made an explicit rule to avoid PowerPoint presentations. There are many games, activities, and ideas available (see https://yparhub.berkeley.edu) that can be adapted to the particular goals of a project. One over-arching approach we have found to be useful in planning activities is beginning with individual or small-group activities or games and then having larger group discussions. This approach serves the dual purpose of breaking the ice by easing into the process and allows for everyone to participate, even if they may not be comfortable sharing with a larger group. Hands-on activities that we used in the infographic development process included adapted free-listing activities, pile sorting activities, marking up and highlighting the most important findings from policy briefs, group voting on the main findings to include, brainstorming and sketching ways to depict findings, and group deliberations and debate. Throughout these processes, it was evident that the students felt ownership and engagement with the process.

We have found that healthy differences in opinions and mild disagreement can be evidence of buy-in to the process that should be celebrated. For example, different groups had strong opinions about what the infographic title should be. Therefore, we adapted the workshop on-the-fly and gave each team a chance to “pitch” their title and explain the rationale. In the end, teams came closer to an agreement on the title, and we made one minor change to incorporate aspects of both into the final title. We found that disagreements, if handled properly, are a fruitful opportunity to have meaningful discussion and debate that reinforces critical thinking, conflict resolution, and leadership skills among the youth.

### Embracing Creativity

Setting up some parameters early is important to maintain focus on the audience and purpose, but it is equally important not to stifle creativity and to let youth drive the process. Many of the previous lessons learned (e.g., flexibility, allowing time, multiple ways to participate) that we have described help to facilitate creativity. For example, many of the best and creative visual ideas came out of the group-based, hands-on activities where we gave few other instructions than “draw some ways to show the findings.” At this stage, it is important to give creative freedom and reiterate that the purpose of the activity is to generate and brainstorm ideas, while also setting clear expectations that not all ideas will fit into the final product. The drawings produced by the youth served as the building blocks and inspiration for the overall style the final infographic would take.

Embracing cultural background and identities is also an important aspect of creativity. In the process of developing the electronic version of the infographic, some of the youth emphasized their vision of using bright and colorful palettes. They cited their stylistic inspiration as *papel picado* (cut paper), a colorful decoration common in *Dia de los Muertos* (Day of the Dead) celebrations in Mexico and the US. The result was a bright color scheme that served as a subtle reminder of the cultural heritage of Latine child farmworkers.

Finally, nourishing creativity beyond the development of a single infographic can yield impressive new directions. For example, we used the format, bright colors, and style of the infographic to develop additional infographics (with the continued feedback of new cohorts of *Levante* program youth) (Quandt et al., 2023). Using the infographic as the starting point, later cohorts of *Levante* program developed additional creative ways to share and adapt the infographics including popular theater workshops and rewriting lyrics to culturally relevant songs to include themes relating to heat stress. These examples demonstrate how embracing creativity can spur additional dissemination strategies driven by youth.

## Conclusion

Partnering with youth can be a powerful way to disseminate research findings. The process we co-developed with youth for infographics could be adapted to a variety of interdisciplinary, community-engaged research settings and may result in unique dissemination strategies. Infographics are one way to disseminate research findings, but many other tools are available. The key aspect is to let youth drive the process. Several years after their release, the infographics continue to be resources that have been used and re-shared during statewide and national campaigns seeking to improve the health and safety of adult and child farmworkers.

## Supplementary Material

1

## Figures and Tables

**Figure 2:**