# Supplementary material for: Disseminating Research Results to Raise Awareness About Child Farmworker Health Through Youth-Driven Participatory Infographics
Source: J Particip Res Methods. Author manuscript; Available in PMC 2026 Jul 18. (PMC13379155; doi:10.35844/001c.159444)
Supplement: 1 [file NIHMS2189364-supplement-1.pdf]

Supplemental Material: Additional Infographics

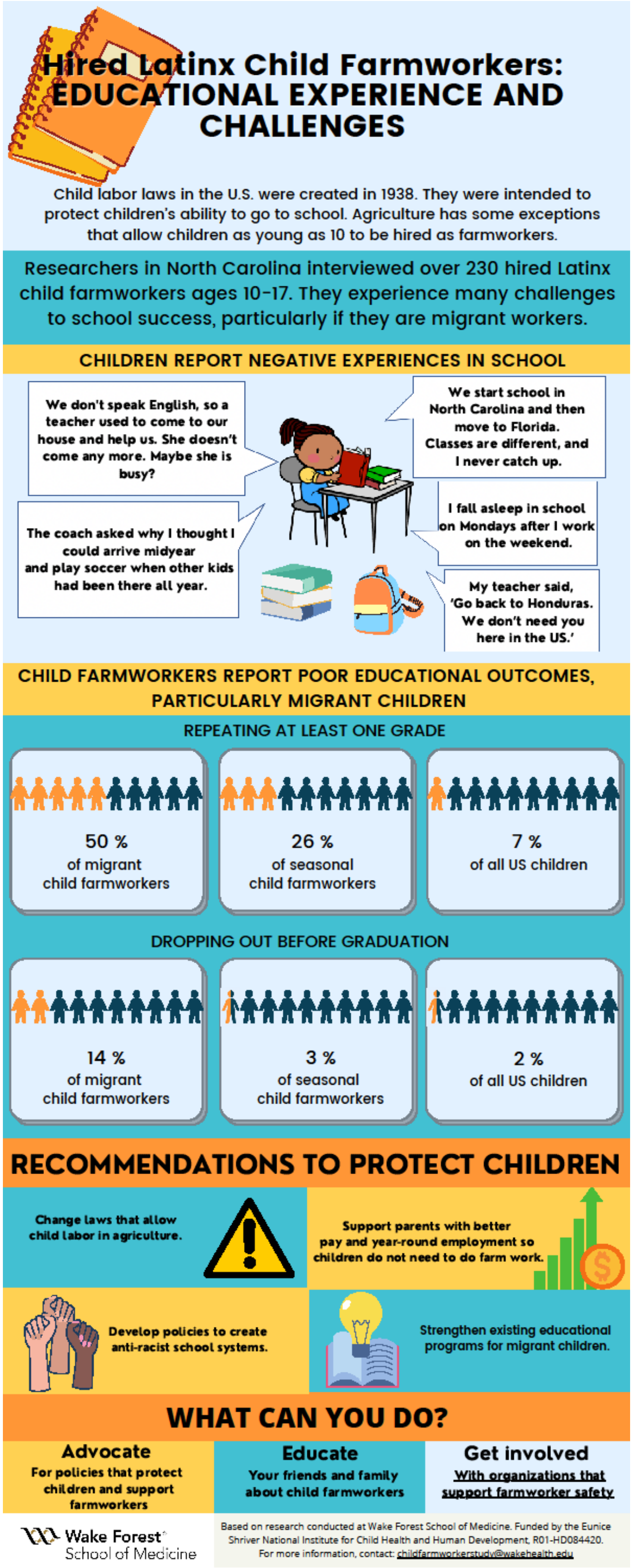

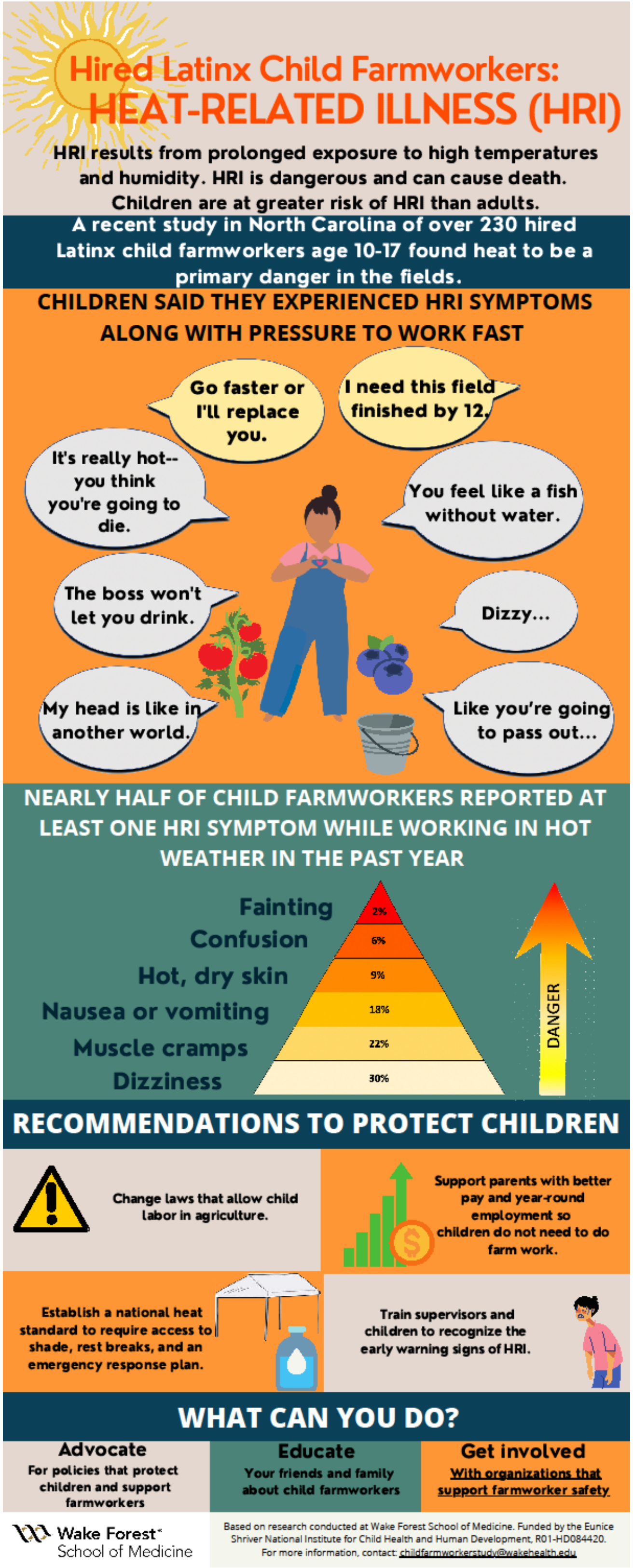
Hired Latinx Child Farmworkers:
HEAT-RELATED ILLNESS (HRI)
HRI results from prolonged exposure to high temperatures and humidity. HRI is dangerous and can cause death. Children are at greater risk of HRI than adults.
A recent study in North Carolina of over 230 hired Latinx child farmworkers age 10-17 found heat to be a primary danger in the fields.
CHILDREN SAID THEY EXPERIENCED HRI SYMPTOMS ALONG WITH PRESSURE TO WORK FAST
Go faster or I'll replace you.
I need this field finished by 12.
It's really hot-- you think you're going to die.
You feel like a fish without water.
The boss won't let you drink.
Dizzy...
My head is like in another world.
Like you're going to pass out...
NEARLY HALF OF CHILD FARMWORKERS REPORTED AT LEAST ONE HRI SYMPTOM WHILE WORKING IN HOT WEATHER IN THE PAST YEAR
Fainting 2%
Confusion 6%
Hot, dry skin 9%
Nausea or vomiting 18%
Muscle cramps 22%
Dizziness 30%
DANGER
RECOMMENDATIONS TO PROTECT CHILDREN
Change laws that allow child labor in agriculture.
Support parents with better pay and year-round employment so children do not need to do farm work.
Establish a national heat standard to require access to shade, rest breaks, and an emergency response plan.
Train supervisors and children to recognize the early warning signs of HRI.
WHAT CAN YOU DO?
Advocate
For policies that protect children and support farmworkers
Educate
Your friends and family about child farmworkers
Get involved
With organizations that support farmworker safety
Wake Forest School of Medicine
Based on research conducted at Wake Forest School of Medicine. Funded by the Eunice Shriver National Institute for Child Health and Human Development, R01-HD084420.
For more information, contact: childfarmworkerstudy@wakehealth.edu

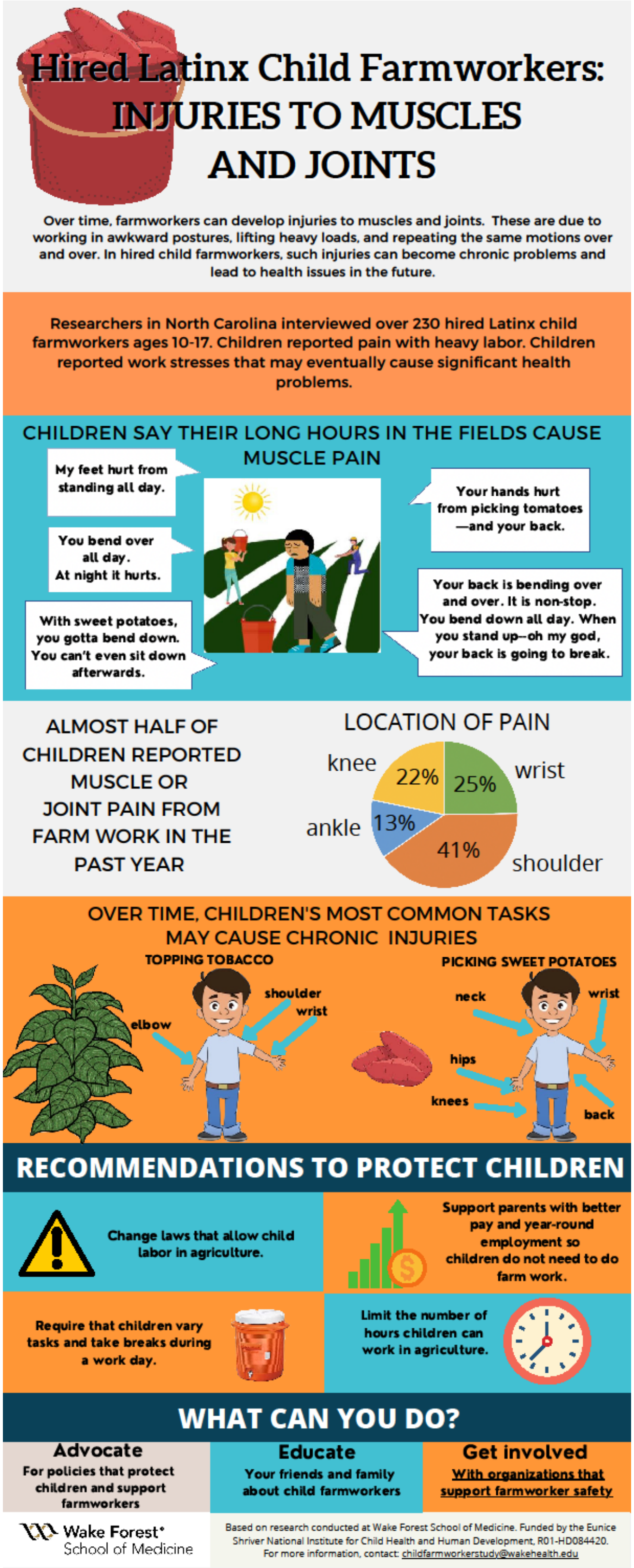
Hired Latinx Child Farmworkers: INJURIES TO MUSCLES AND JOINTS
Over time, farmworkers can develop injuries to muscles and joints. These are due to working in awkward postures, lifting heavy loads, and repeating the same motions over and over. In hired child farmworkers, such injuries can become chronic problems and lead to health issues in the future.
Researchers in North Carolina interviewed over 230 hired Latinx child farmworkers ages 10-17. Children reported pain with heavy labor. Children reported work stresses that may eventually cause significant health problems.
CHILDREN SAY THEIR LONG HOURS IN THE FIELDS CAUSE MUSCLE PAIN
My feet hurt from standing all day.
You bend over all day. At night it hurts.
With sweet potatoes, you gotta bend down. You can't even sit down afterwards.
Your hands hurt from picking tomatoes —and your back.
Your back is bending over and over. It is non-stop. You bend down all day. When you stand up—oh my god, your back is going to break.
ALMOST HALF OF CHILDREN REPORTED MUSCLE OR JOINT PAIN FROM FARM WORK IN THE PAST YEAR
LOCATION OF PAIN
knee 22%
wrist 25%
ankle 13%
shoulder 41%
OVER TIME, CHILDREN'S MOST COMMON TASKS MAY CAUSE CHRONIC INJURIES
TOPPING TOBACCO
shoulder
wrist
elbow
PICKING SWEET POTATOES
neck
wrist
hips
knees
back
RECOMMENDATIONS TO PROTECT CHILDREN
Change laws that allow child labor in agriculture.
Support parents with better pay and year-round employment so children do not need to do farm work.
Require that children vary tasks and take breaks during a work day.
Limit the number of hours children can work in agriculture.
WHAT CAN YOU DO?
Advocate
For policies that protect children and support farmworkers
Educate
Your friends and family about child farmworkers
Get involved
With organizations that support farmworker safety
Wake Forest® School of Medicine
Based on research conducted at Wake Forest School of Medicine. Funded by the Eunice Shriver National Institute for Child Health and Human Development, R01-HD084420.
For more information, contact: childfarmworkerstudy@wakehealth.edu

Supplementary Materials

Additional infographic 1

Download: https://jprm.scholasticahq.com/article/159444-disseminating-research-results-to-raise-awareness-about-child-farmworker-health-through-youth-driven-participatory-infographics/attachment/336566.pdf

---

Additional infographic 2

Download: https://jprm.scholasticahq.com/article/159444-disseminating-research-results-to-raise-awareness-about-child-farmworker-health-through-youth-driven-participatory-infographics/attachment/336565.pdf

---

Additional infographic 3

Download: https://jprm.scholasticahq.com/article/159444-disseminating-research-results-to-raise-awareness-about-child-farmworker-health-through-youth-driven-participatory-infographics/attachment/336564.pdf

---

Figure 1

Download: https://jprm.scholasticahq.com/article/159444-disseminating-research-results-to-raise-awareness-about-child-farmworker-health-through-youth-driven-participatory-infographics/attachment/336567.pdf

---

Original infographic

Download: https://jprm.scholasticahq.com/article/159444-disseminating-research-results-to-raise-awareness-about-child-farmworker-health-through-youth-driven-participatory-infographics/attachment/336568.pdf

---
